# Use of ultrasonography in the evaluation of patients with sleep apnea: a systematic review

**DOI:** 10.1016/j.bjorl.2024.101468

**Published:** 2024-07-14

**Authors:** Leticia Andrade de Angelo, Fernando Linhares Pereira, Bruno Bernardo Duarte, Michel Burihan Cahali

**Affiliations:** Hospital das Clínicas da Faculdade de Medicina da Universidade de São Paulo, São Paulo, SP, Brazil

**Keywords:** Elastography, Obstructive sleep apnea, Pharynx, Tongue, Ultrasound

## Abstract

**Objectives:**

This study aims to review the current role of various ultrasonographic methods in the evaluation of the upper airway in patients with obstructive sleep apnea.

**Methods:**

A literature review was performed on the medical databases: Pubmed, Web of Science, Scopus and Embase. After analyzing the available studies, six of them were selected for data extraction.

**Results:**

All selected studies demonstrated that it is possible to use ultrasonography as a method of evaluating the upper airway. Studies with gray-scale ultrasound concluded that the lateral pharyngeal wall and tongue are thicker in patients with obstructive sleep apnea compared to non-apneic patients. Moreover, studies with tissue characterization ultrasound, nowadays called quantitative ultrasound, have identified unique features in obstructive sleep apnea patients: standardized backscatter ultrasonography demonstrated that this particular sound wave analysis is associated with the severity of obstructive sleep apnea. Ultrasonography with elastography shows that the electrical stimulus generated in the hypoglossal nerve results in greater stiffness on the side of the tongue that is stimulated; whereas studies show conflicting results regarding the evaluation of baseline tongue stiffness in obstructive sleep apnea patients compared to non-apneic subjects.

**Conclusion:**

There is feasibility of different methods of ultrasonographic evaluation of the upper airway, with emphasis on ultrasonographic methods of tissue characterization, such as elastography, which proved to be a promising method of evaluating the mechanical properties of the muscles involved in the pathogenesis of obstructive sleep apnea and which require further studies for a better elucidation of its applicability.

## Highlights

• Ultrasonography may be a promising new method of assessing obstructive sleep apnea.
• Lateral pharyngeal wall is a thicker structure in apneic patients.
• Elastography allows measuring the tissue stiffness of the airways in apneics.
• Ultrasonography demonstrated measures associated with the severity of sleep apnea.

## Introduction

The pathophysiology of Obstructive Sleep Apnea (OSA) is multifactorial and not fully understood. We know, however, that several factors influence it, such as anatomical characteristics, reduction in the threshold for awakening, inadequate ventilatory control and insufficient muscular responses. Thus, several studies have sought to contribute to the elucidation of its etiopathogenesis.

With the marked reduction in muscle tone that occurs during sleep, patients with snoring and OSA have excessive mobility of the pharyngeal tissues, causing vibration and recurrent collapses in the Upper Airway (UA).^1^ As the reduction of this muscle tone is a phenomenon inherent to the natural sleep of humans, several studies have investigated possible tissue alterations in the UA of patients with OSA that could contribute to the loss of UA support during sleep.

Some studies have shown alterations in the aspect of the palatopharyngeal muscle fibers in apneic patients, such as changes in the normal diameter of the fibers and histological signs of neurogenic lesions (nuclear alterations and signs of fiber regeneration) and concluded that these alterations were due to the vibratory trauma of snoring, present both in the group of apneic and non-apneic snorers.^2^, ^3^, ^4^ Dantas et al. showed an increase in type I collagen in the extracellular matrix of the superior pharyngeal constrictor muscle in apneic snorers compared to non-apneic non-snorers.^5^

The MRI can show the dynamic behavior of UA tissues during sleep but, in general, it does not provide data on the mechanical properties of those tissues, such as hardness or flexibility.^6^ Passos et al. found, using Computed Tomography (CT), that upper airway narrowing during sleep in apneics occurs predominantly in the retropalatal region and affects the anteroposterior and lateral dimensions, in addition to being related to sleep-dependent thickening of the Lateral Pharyngeal Walls (LPW) and posterior displacement of the tongue.^7^

Schotland et al. used MRI to evaluate the upper area of patients with apnea and observed that the genioglossus and geniohyoid muscles are thicker in this group when compared to the control group.^8^

Schwab et al., using MRI, demonstrated that the volume of the tongue and lateral pharyngeal wall are greater in apneic individuals when compared to controls and, through a multivariable logistic regression, that they are independent risk factors for apnea of sleep.^9^

Despite the usefulness of CT and MRI scans, we are often faced with some limitations. MRI is expensive, restricts the patient’s weight, can cause claustrophobia, and takes a long time to acquire images. CT, on the other hand, also has restrictions regarding the patient’s weight, in addition to generating exposure to high levels of radiation.

The diagnostic Ultrasound exam (USG) uses the emission of high frequency acoustic (sound) pulses to acquire echo data that are processed into medical images. In recent decades, it has been widely used to assess musculoskeletal disorders. Because it is an innocuous, non-invasive test, without the emission of ionizing radiation, portable, easily available and relatively low cost, this diagnostic tool presents itself as a viable alternative possibility in the evaluation of muscles involved in OSA.^10^, ^11^

Using the different types or modalities (called modes) of ultrasound, we can evaluate: with the B mode (brightness), the morphology of the structures; with the Doppler mode, tissue vascularization; and with elastography, the mechanical properties of tissues (elasticity). This last mode is one of the ways to obtain tissue characterization using ultrasonography.

Elastography is an emerging technology used in the non-invasive and quantitative assessment of soft tissue elasticity.^12^, ^13^ It assesses tissue stiffness, fundamentally by tracking specific acoustic wave velocities (shear waves) that propagate along the plane perpendicular to the wave pulse emitted by the transducer. Ultrasound equipment, used in daily clinical practice, infers tissue elasticity by calculating the Young’s modulus (E = 3 dVs^2^; where “E” is elasticity, “d” is the density of the medium and “Vs^2^” is the square of the velocity of the shear waves), where it is considered that the higher the speed of the shear waves (which translates into a higher elastography value), the greater the stiffness of the tissue, with a greater correlation with some pathological states (atherosclerosis, neoplasms, fibrosis, etc.). Several applications of this modality are already in use in daily practice, such as the elastographic study for classifying the degree of liver fibrosis, which avoids the need for liver biopsies, reducing the risk of complications and the cost of treatment.

Tissue characterization of the upper airway using elastography associated with MRI was described a few years ago.^14^ A study of nine patients with OSA demonstrated that tongue hardness is 10% lower in these patients compared to controls without OSA.^15^ The explanation for this may lie in the increase in fatty infiltration at the base of the tongue in patients with OSA.^16^

Ultrasonography for tissue characterization, which is now called quantitative ultrasound, has recently been introduced in the evaluation of OSA.

The aim of this review is to establish the status of ultrasound imaging and quantitative ultrasound in the evaluation of patients with OSA, with a focus on the role of ultrasound with elastography.

## Methods

Using as a guide the question “Are there differences in the anatomy and elastographic properties of the airway of apneic patients compared to non-apneic patients when evaluated by USG?”, the research followed the acronym PICO, where “P” corresponds to population/patients, “I” for intervention, “C” for comparison or control and “O” for outcome which, in English, means clinical outcome, being then, P — patients with obstructive sleep apnea; I — elastography values; C — comparison with control group; O — elastographic differences in airway structures.

The literature review was performed using databases: Pubmed, Web of Science, Scopus and Embase.

In the search, the terms in English were used: “upper airway” and “Sleep Apnea Obstructive” or “Apnea, Obstructive Sleep” or “Apneas, Obstructive Sleep” or “Obstructive Sleep Apnea” or “Obstructive Sleep Apnea Syndrome” or “Obstructive Sleep Apneas” or “Sleep Apnea Hypopnea Syndrome” or “Sleep Apnea Syndrome, Obstructive” or “Sleep Apneas, Obstructive” or “Syndrome, Obstructive Sleep Apnea” or “Syndrome, Sleep Apnea, Obstructive” or “Syndrome, Upper Airway Resistance, Sleep Apnea” or “Ultrasound shear-wave elastography” or “MR elastography” or “Ultrasound” or “Sonoelastography”.

We included prospective observational studies that used ultrasound in any of its modes as a method for evaluating upper airway tissues in patients with OSA compared to normal subjects. Studies were excluded through analysis of title and abstract and that were in the format of a narrative review or systematic review article, as well as studies to evaluate the property of cervical vessels, infectious, inflammatory or tumor processes of the upper airways. The comprehensive period did not have a time limit or age limit for the individuals included in the studies. The studies were evaluated and selected by two investigators.

The following data were collected from the studies: title; author; year of publication; name of publication journal; key words; study design; age; sex; sample size; type of exam used in the evaluation; structures evaluated in the upper airway; values obtained after the analysis of the structures; comparison of values between evaluated groups; influence of the values obtained in the AOS.

## Results

**Figure 1:**
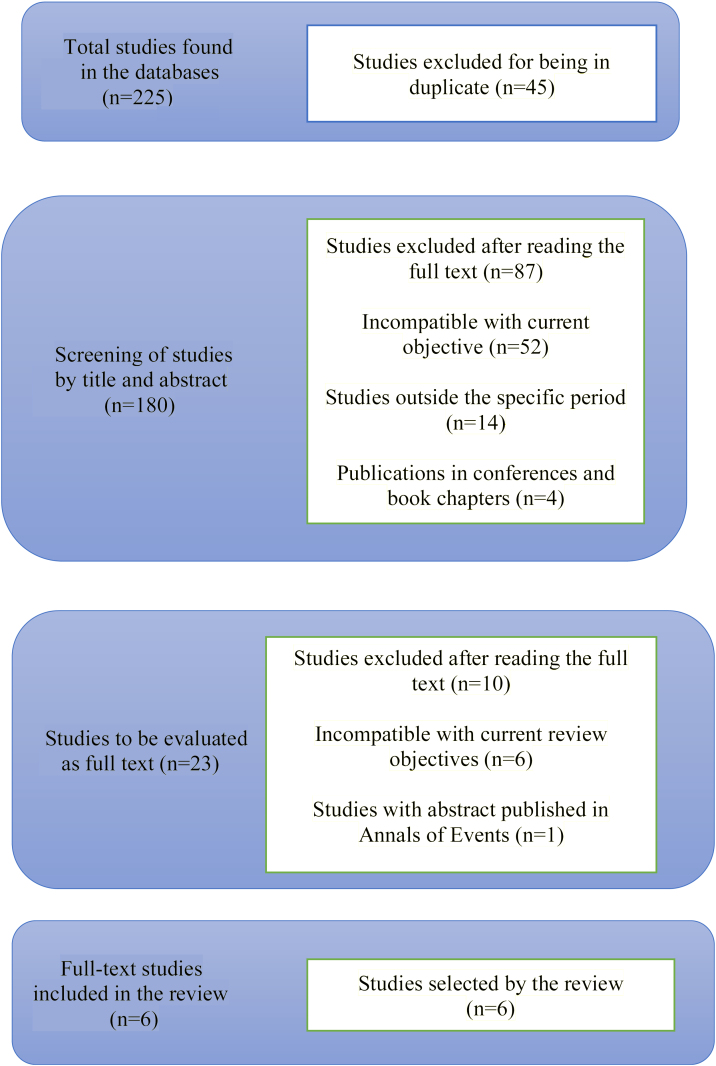
Flowchart of the selection process of studies included in the review.

Searches in the aforementioned databases were carried out until 04/26/2023 and resulted in 225 articles, as follows: Pubmed (title, subject and abstract) — 96; Web of Science (title, subject and abstract) — 82; Scopus (title, subject, abstract) — 47. After excluding articles in duplicate, 180 articles remained. A total of 157 articles were also excluded by the author after reading the title and abstract, totaling 23 studies to be reviewed by the authors. After searching for the full-text articles, only six studies met the inclusion criteria (Fig. 1).

Data extracted from the articles included in the systematic review are summarized in Table 1.

**Table 1:** General data of selected studies per year of publication.

Author/year	Level of scientific evidence/type of study/scientific evidence	n	Evaluation method (evaluated structure)	Main findings	Limitations
Liu et al.^17^; 2007	Prospective observational	76	B mode USG of the neck (parapharyngeal space)	LPW thickness greater in OSA group	Association of the fat deposit with OSA could not be evaluated
	Case — control: 3b			Positive and independent correlation between AHI and LPW thickness in OSA group	Extension of the neck during the examination may affect the diameter of the airway
Lin et al.^18^; 2018	Prospective observational	82	B mode USG of the neck (parapharyngeal space and its structures)	LPW thickness and total neck thickness greater in OSA group	Small sample size
					Different positions of the neck during US examination and sleep
	Case – control: 3b			Positive correlation between the total thickness of the LPW and the AHI values	Impossibility of evaluation of fat deposition in the cervical region
Chang et al.^21^; 2020	Prospective observational	46	USG elastography of the neck (tongue)	Higher values of elastography in OSA group in the entire tongue extension	Characteristics of the participants not equally distributed in the two group
	Case — control: 3b			Elastography values in the middle third of the tongue indentified as independent predictor for the presence of OSA	OSA group was older, had a higher BMI and larger neck circumference than controls
				Tongue stiffness greater in OSA group	No gold standard regarding tongue stiffness is referenced in this study
Chu et al.^22^; 2021	Prospective observational	69	USG elastography of the neck (tongue)	Thicker tongues in OSA group	Healthy controls did not receive PSG examinations
	Case — control: 3b			No significant difference in tongue stiffness between the two groups (sagittal plane)	Case number relatively small
				Increase in tongue stiffness in the control group (coronal plane)	
				Risk of OSA was inversely associated with the coronal assessment of tongue muscle stiffness – softer tongue in group OSA	
Arens et al.^20^; 2021	Prospective observational	18	USG elastography of the neck (tongue)	Increase in stiffness of the tongue during stimulation of the muscles ipsilateral to the implant in relation to the muscles on the non-implanted side	Small total study population
	Case — control: 3b				Small number of female patients
					Large number of stimulators implanted on the right side
Lui et al.^19^; 2023	Prospective observational	89	Backscattered USG of the neck (tongue base)	Backscatter was able to significantly differentiate patients with AHI < 15 from those with AHI ≥ 15	Small sample size
	Case — control: 3b				Single-institution experience
				Moderate association between backscatter and the AHI	Underrepresentation of the female sex, and unequal racial and ethnic distribution

### B-mode ultrasonography in the evaluation of OSA

Liu et al.^17^ conducted a study that analyzed, using USG, measurements of the thickness of the lateral pharyngeal wall of apneic patients and compared these measurements with the AHI. Seventy-six patients with suspected OSA were selected, who underwent Polysomnography (PSG), dividing the group into 58 apneic and 18 non-apneic, and subsequently evaluated by USG examination in the supine position with a transducer positioned longitudinally on the side of the neck, below of the lateral edge of the occipital bone, analyzing the parapharyngeal space. In this study, there was a positive and independent correlation between the AHI and the lateral pharyngeal wall thickness measured on USG, as well as strong evidence that the lateral pharyngeal wall thickness is greater in apneic patients when compared to non-apneic ones.

Another study, carried out Lin et al.^18^ aimed to use USG to evaluate upper airway structures in children and compare the measurements of these structures with the severity of OSA, previously diagnosed by PSG. The studied group consisted of 82 children younger than 18 years old, with indication for adenotonsillectomy, 20 of them primary snorers (AHI < 1) and 62 apneic (AHI > 1).

The maximum size of the tonsils (Width [W] and Length [L]), the distance from the skin to the surface of the tonsils (Tonsillar Neck Thickness — TNT) were measured, and the tonsil/neck ratio was calculated, defined by the proportion of the tonsil width in relation to the neck width — W/TNT × 100%), in addition to lateral pharyngeal wall thickness (measurement from the internal carotid artery to the echogenic surface of the pharynx) and parapharyngeal cervical thickness (measurement from the skin to the echogenic surface of the pharynx). There was no statistical difference between primary snorers and OSA regarding tonsillar volume, as well as the tonsil/neck ratio. However, the thickness of the lateral pharyngeal wall at the retropalatal level was significantly greater in the apneic group compared to primary snorers. Total neck thickness was also significantly greater in apneic children compared to primary snorers. They concluded that there was no relationship between tonsillar volume or the mean tonsil-neck ratio with the AHI, while there was a positive correlation between the total thickness of the lateral pharyngeal wall and the AHI values. After multiple linear regression analysis, they conclude that neck and lateral pharyngeal wall thickness are independent risk factors for OSA in children.

### USG by backscatter in the evaluation of OSA

Part of the ultrasound wave collides with the molecules of the studied tissue, creating wave scattering. Part of these scattered waves reach the USG transducer and can be studied and quantified as backscattered waves. In theory, this mode of quantitative ultrasound can identify features of tissue microstructure and identify patterns of abnormality. A recent study by Liu et al.^19^ used backscatter USG to study the tongue base of patients with suspected OSA.

Eighty-nine patients previously evaluated by PSG were selected; 18 (20%) had no OSA, 35 (39%) had mild OSA, 20 (22%) had moderate OSA, and 16 (18%) had severe OSA. The patients were placed in the supine position, on a submental ultrasound scanner positioned in a standardized way (as variations in the angle of the transducer produce changes in this type of exam). When analyzing 4 tongue base regions, they concluded that backscatter was able to significantly differentiate patients with AHI < 15 from those with AHI ≥ 15, and data from two tongue base regions (among the four analyzed) showed a moderate association between backscatter and the AHI.

### USG with elastography in the evaluation of OSA

Study carried out by Arens et al.^20^ evaluated, using USG elastography, the responses caused in the muscles of the upper airway after selective stimulation of the hypoglossal nerve with a hypoglossal nerve implant used in the treatment of OSA. The aim of the study was to investigate whether USG elastography would be able to detect changes in tongue muscle hardness after selective stimulation of the hypoglossal nerve. For this, the stiffness of the geniohyoid and genioglossus muscles on the implanted and non-implanted sides were measured, both with and without stimulation. The study included 18 implanted patients, placed in the supine position, in hyperextension of the neck, with the transducer positioned in the submandibular region in the longitudinal plane and, subsequently, in the transverse plane, and elastographic measurements were obtained for both muscles, bilaterally (side with and without stimulator), with and without hypoglossal nerve stimulation. The results found an increase in stiffness during stimulation of the muscles ipsilateral to the implant in relation to the muscles on the non-implanted side.

Two studies compared elastographic measurements of the tongue in patients with and without OSA, using USG in the coronal and sagittal planes, as mentioned below.

Chang et al.^21^ compared the stiffness of the tongue of 20 control patients and 26 patients with OSA (the two groups with conditions previously confirmed by PSG) with USG elastography. Both groups were evaluated by an otorhinolaryngologist specialized in USG of the head and neck region ‒ hyoid bones) and sagittal (transducer between the hyoid bone and the mandibular symphysis).

Initially, images were acquired in the sagittal plane, in B-mode and six regions of 1.5 cm in diameter each were selected, two in each region of the tongue (anterior, middle and posterior) and elastography was applied in each location with subsequent calculation of the median of each region. Sequentially, images were acquired in the coronal plane, in B-mode, and four other circular regions of 1.5 cm in diameter were selected and elastography was applied with calculation of their median. After analyzing and calculating the medians, the results obtained were in the sagittal plane, the AOS group had significantly higher values than the control group in the entire tongue extension (anterior, middle and posterior thirds), (median 35.1 kPa vs. 19.1 kPa, *p* < 0.0001). In the coronal plane, the AOS group also had significantly higher values than the control group (median 13.4 kPa vs. 10.5 kPa, *p* = 0.011). After adjusting for age, gender, body mass index and neck circumference, elastography values in the middle third of the tongue were identified as an independent predictor for the presence of OSA (Odds Ratio [OR = 1.25], 95% CI 1.03–1.51, *p* = 0.027). It was concluded that tongue rigidity was shown to be greater in patients with OSA compared to controls without OSA during normal breathing.

In another study carried out by Chu et al.,^22^ 37 control patients (assessed and excluded OSA by the STOP questionnaire)^23^ and 32 patients with OSA previously diagnosed by PSG were selected. Both groups underwent ultrasound analysis in the supine position, with neck hyperextension to expose the submental region, where the transducer was located and tongue thickness measurements were obtained (distance between the deep fascia of the geniohyoid muscle and the point of greatest thickness of the dorsum of the tongue) in the sagittal plane (transducer positioned between the hyoid bone and the mandibular symphysis) and coronal plane (transducer parallel to the anterior portion of the hyoid bone) and elastography was applied in kPa units in 5 circular regions in the center of the tongue, in both plans mentioned above. In univariate analysis, OSA was associated with increased tongue thickness in the sagittal (OR = 1.15; 95% CI 1.04–1.27) and coronal (OR = 1.21; 95% CI 1.09–1.34 planes). Therefore, patients with OSA exhibited significantly thicker tongues in both the sagittal and coronal planes. There was no significant difference in tongue stiffness between the two groups in measurements in the sagittal plane (*p* = 0.61), while in the coronal plane there was an increase in tongue stiffness in the control group (from 14.05 ± 4.01 vs. 11.34 ± 1.97 kPA, *p* = 0.006). After adjusting for age, gender, neck circumference, and body mass index, there was an increase in tongue muscle thickness (OR = 1.16; 95% CI 1.01–1.32) in patients with OSA, as well as how the risk of OSA was inversely associated with the coronal assessment of tongue muscle stiffness (OR = 0.72; 95% CI 0.54–0.95). There were no significant differences in tongue stiffness between OSA patients of different disease severity (OR = 0.98; 95% CI 0.89–1.07).

## Discussion

The six studies presented show us that it is feasible to perform USG as a method of evaluating the upper airway, both in the B-mode, which provides us with data on morphology, and in the elastography mode, capable of measuring the stiffness of the evaluated tissues, and in the backscatter mode, which characterizes the tissue microstructure.

Three of the cited studies showed that there are significant morphological differences in the upper airway of patients with OSA. Two of them evaluated the lateral pharyngeal wall and concluded that it is thicker in apneic patients compared to non-apneic ones.^17^, ^18^ The third study,^22^ which evaluated the tongue of patients, also concluded that it was thicker in both assessed planes (coronal and sagittal) when comparing apneic and non-apneic patients.

One single study used standardized US by backscatter^19^ and demonstrated that it is feasible to assess the upper airway of patients using this method and that the values obtained are associated with the severity of OSA.

One of the studies that used elastography as an evaluation method concluded that the electrical stimulus generated in the hypoglossal nerve results in greater rigidity of the innervated muscles when compared to muscles and nerves that are not stimulated.^20^

However, two of the included studies showed conflicting results. In the study by Chang et al.,^21^ who used elastography to evaluate the patients’ tongue, found greater lingual rigidity in apneic patients when compared to non-apneic ones in both evaluated planes (coronal and sagittal), while in the study by Chu et al.,^22^ using the same evaluation method, the results were lower tongue rigidity in the coronal plane in apneic patients compared to non-apneic patients, and no difference between groups in measurements in the sagittal plane of the tongue. Differences in results between the two studies could be attributed to factors such as differences in fat deposition at the base of the tongue in obese and elderly patients,^24^ as well as age differences between participants, and in the first of them^21^ there was a lower mean age in the control group compared to the apneic group, while in the second study^22^ there was no significant difference between the ages of the compared groups.

Despite the cited studies having a limited number of participants and in some of them presenting conflicting results, all of them pointed to the existence of differences in the morphological and functional properties of the upper airway of patients with OSA and that require further research to be in fact elucidated. As USG is a very safe and relatively inexpensive and widespread method, its applicability in the evaluation of patients with suspected OSA appears to be promising.

## Conclusion

Currently, we have a vast arsenal of methods for evaluating the upper airway, as mentioned in this review. Most of them still have their clinical applicability limited by the lack of studies that prove their accuracy, either for the diagnosis of OSA, as well as for monitoring its evolution.

There is feasibility of different methods of ultrasonographic evaluation of the upper airway, with emphasis on ultrasonographic methods of tissue characterization, such as elastography, which proved to be a promising method of evaluating the mechanical properties of the muscles involved in the pathogenesis of OSA and which requires further studies to determine its clinical usefulness.

## Funding

Michel Burihan Cahali financial investor on Biologix company.

## Conflicts of interest

The authors declare no conflicts of interest.
